# New Work and Its Problems at Ipswich

**Published:** 1915-08-21

**Authors:** 


### THE WAR'S EFFECT ON AN EAST COAST HOSPITAL.

### New Work and its Problems at Ipswich.

A RECORD of what can be achieved by a united effort, even in a district which has suffered industrially and financially by the war, when a policy has once been decided on, is contained in the seventy-ninth annual report of the East Suffolk and Ipswich Hospital. It is of unusual interest. The statement of accounts for the financial year ended May 31 last, which has recently been presented to the Anniversary Court of Governors of that institution illustrates the above contention.

Some ten years ago the hospital was in a state when an annual revenue expenditure of £7,000 entailed a yearly deficit of £400 or £500. From this lethargic state it was awakened by some energetic members of its managing body, and in 1913 structural alterations and improvements had just been completed which had cost over £20,000 and necessitated an increase in the annual expenditure of over £2,000.

As to the financial side of the hospital's work a special appeal was apparently issued for funds to enable the number of beds to be increased from 143 to 260. £3,000 was asked for, and within a few days the sum realised was £5,083, the balance of which is being devoted to the new scheme for a further 120 beds. In regard to the revenue account for the year, the expenditure was £348 more than the income, but as one of the statutes enacts that all legacies over £100 shall be transferred to capital, a sum of £200 was excluded from the revenue account, otherwise the deficit is really only £148 on a revenue expenditure of £11,460.

In the Report in THE HOSPITAL (1913) the opinion was expressed that the income should be increased by some £2,000. It is now to be noted with satisfaction that the amount of the annual subscriptions for the year is the highest on record, being £828 more than it was in 1913. Unfortunately the shutting down of works and the loss of a large number of men, who have gone to the Front, have occasioned a somewhat serious drop in the employees' contributions, the organisation for which has recently been a feature in the Ipswich Hospital accounts.

The men who could meet the need for such an expenditure, when the financial outlook appeared so much in need of improvement, arranged, on the day following the outbreak of hostilities, for this hospital to be made ready for the reception of the wounded men of the Navy. When it was found that the Army had more immediate need of hospital accommodation than the Navy the offer was transferred, and since then convoys of wounded have been sent direct to the East Suffolk Hospital from the embarkation departments. To facilitate the reception of wounded in large numbers one of the vice-presidents, Mr. Wm. Paul, fitted up his house and handed it over to the Order of St. John of Jerusalem for the reception of part of the convoys, for which the East Suffolk Hospital had not then sufficient accommodation. This hospital was also voluntarily staffed by members of the hon. medical staff of the East Suffolk and Ipswich Hospital.

To enable the hospital to deal efficiently and expeditiously with its wounded, schemes had to be devised for opening a number of private convalescent homes in that part of the county, and this difficult problem was successfully tackled and solved with the hearty co-operation of private individuals and the aid of the British Red Cross Society and the Order of St. John of Jerusalem. The hospital has remained throughout responsible for the patients transferred, inasmuch as the records, official forms, and correspondence are transacted in the secretary's department of the central institution, under Mr. Arthur Griffiths.

The various steps taken by the board of management to provide accommodation for the extra work undertaken are dealt with in the report. Briefly, the original nurses' building (which, incidentally, contained the cubicles on which adverse comments were made in THE HOSPITAL, December 6, 1913, page 253) has been reconstructed internally and turned into wards for children, the previous cubicles having been swept away. The Pamela Cobbold balconies, and the wards to which they are attached, have been allocated entirely to the wounded men; and the patients who have the opportunity of breathing the pure air of the high land upon which the hospital stands in these open-air wards are in an enviable position. The extension of this open-air balcony system to all the main wards, through the generosity of one gentleman, has begun by a small open-air balcony for the use of the wounded men in the Goodrich Ward.

The hospital has had the advantage of having a worthy chairman in Mr. John Dupuis Cobbold, the Mayor of Ipswich, a gentleman whose family for generations has been one of the mainstays of the hospital, and who himself has spared neither time nor money for its welfare. He was one of the pioneers of the open-air ward system, and gave the hospital the Pamela Cobbold balconies.

Further alterations have been made to the hospital internally, and an extension has been carried out by a substantial, although it is termed temporary, ward of 46 beds. These additions increased the accommodation of the hospital from 143 to 260 beds, all of which save 46 are of a permanent character. When this scheme was completed, the Board were not fully satisfied that they had done everything that might be expected of them. As the Report states: "Even with these additions the Board felt that the accommodation for wounded was not sufficient for a town like Ipswich, and after every expedient in the way of hiring and adapting other buildings in the locality had been discussed and negatived by the authorities, it resolved to launch out still further and to offer the War Department up to 200 extra beds for the wounded in temporary buildings to be erected on the ground of the Ipswich School adjacent to the hospital. This offer has been most cordially accepted by the War Office, and at the time of drafting this report a contract has been let for the erection of three wards containing 120 beds in all, with the necessary sanitary conveniences, etc. The board has resolved to name these three buildings the Mason Ward, the Ripley Ward, and the Bartlet Ward, after those gentlemen who have rendered such good service to this institution."

The Board's Attitude to War Work and Salaries.

A casual reader in another part of the country might wonder why the Ipswich people should undertake such a work for the wounded. It seems clear from the context that full use has been made of the hospital for the reception and treatment of serious surgical cases amongst the local troops, while a great responsibility attaches to the board of management in respect of the admission and treatment of civil wounded from any aerial raids as well as from a coast bombardment. The board has obtained the use of two large houses in the immediate vicinity as additional accommodation for nurses, and despite the number of trained sisters and nurses who have left the institution to nurse with the Colours, an adequate nursing staff is being maintained. The management recognises the value of the resident medical staff by increasing emoluments in line with the more generous hospitals, while the members of the nursing staff benefit by a further rise in the standard of their salaries and a special war bonus. The governing body also places a high value on voluntary gifts, whether in coin or kind.

The Value of Lay Helpers.

In the laundry a rota of some ten or a dozen ladies work the steam ironing calender and release for other purposes those members of the paid staff who usually operate this machinery. In the kitchen, ladies have an opportunity of showing their practical skill in cooking. Members of the V.A.D., who have hitherto been under training in the out-patient department only, now assist regularly in the wards, while a squad of men of the R.A.M.C. destined for the Front can be found working and acquiring their practical training in the wards and theatres.

The Hospital Supply Depot.

Any article on the East Suffolk Hospital which gives an account of its war work should make at least a passing reference to the assistance given to that institution by the Ipswich Hospital Supply Depôt—an organisation worked entirely by the ladies of the Borough and having its headquarters at Northgate Street, Ipswich. This depôt has, in addition to equipping some of the new wards in the hospital, supplied requisites for several hospitals abroad, sent dressings and other medical needs to the suffering Serbians, and generally helped at home and abroad.

